# Biosafety of Non-Return Valves for Infusion Systems in Radiology

**DOI:** 10.1038/s41598-020-66491-y

**Published:** 2020-06-12

**Authors:** Marcela Padilha Facetto Azevedo, Rachel Maciel Monteiro, Carla Castelani, Felipe Lazarini Bim, Lucas Lazarini Bim, Ana Paula Macedo, Viviane de Cássia Oliveira, Evandro Watanabe

**Affiliations:** 1 Department of Fundamental Nursing, College of Nursing of Ribeirão Preto, University of São Paulo, Ribeirão Preto, São Paulo Brazil; 2 Department of Restorative Dentistry, School of Dentistry of Ribeirão Preto, University of São Paulo, Ribeirão Preto, São Paulo Brazil; 3 Department of Dental Materials and Prosthodontics, School of Dentistry of Ribeirão Preto, University of São Paulo, Ribeirão Preto, São Paulo Brazil; 4 University of Valley of the Sinos River (Unisinos), São Leopoldo, in the Metropolitan Area of Porto Alegre, Porto Alegre, Rio Grande do Sul Brazil

**Keywords:** Bacteriology, Preventive medicine

## Abstract

Cross-infection in contrast injectors is still a subject under discussion with little understanding. This study evaluated the biosafety of non-return valves (NRVs). Initially, the maximum pressure during backflow of intact and disrupted flexible diaphragms (FDs) from NRVs, as well as the functionality of connectors with NRVs were verified. The performance of air columns interposed by water in connectors with NRVs was analyzed, and the diffusion distance of crystal violet through connectors with NRVs was measured. The efficacy of NRVs as a barrier to bacterial contamination from backflow was evaluated. Finally, a clinical study of bacteriological contamination from syringes was conducted. There were differences among the maximum tolerated pressure by intact and disrupted FDs. Disrupted FDs showed no failures in the functionality of connectors with NRVs based on the lack of air bubbles released. Air columns could move through connectors with NRVs with intact and disrupted FDs. The longest diffusion distance of crystal violet was 6 cm of connector length, and NRVs showed efficacy as a barrier to bacterial contamination. In the clinical study, there was no bacterial growth in any of the evaluated samples. In conclusion, biosafety depends on the functionality of NRVs as well as proper practical clinical performance.

## Introduction

Knowledge of radiology covers specialties such as computed tomography (CT), magnetic resonance imaging (MRI) and digital radiology. Moreover, diagnostic methods enable obtaining information that is increasingly fast, efficient and linked to new technologies^1^. The techniques of these exams are relatively simple to perform, but they require carefully defined protocols, contrast dosages, and injection speeds and times and the need to comply with measures related to biosafety^2^.

In health, non-return valve (NRV) usage has greatly increased in the last decade, so much so that these valves came to be present in surgeries with propofol, in vesical catheters and for urinary tract infection patients^3,4^. For use in health, such valves are designed, but not all are evaluated. CT and MRI employ contrast injectors coupled to syringes and connectors. Such exams have recognized value in the diagnosis of possible pathologies and therapeutic decisions^5,6^. Through contrast injectors, the main purpose of NRVs is to prevent the backflow of blood^7^.

In regard to connectors with NRVs, it is important to consider bloodstream infections (BIs) because approximately 60% of bacteremia cases are associated with some intravascular devices. BI rates range from 2.7 to 11.7 per 1000 catheters/day in the USA, and the Centers for Disease Control and Prevention established recommendations to reduce BI rates^8–11^. Furthermore, healthcare-associated infections (HAIs) are a challenge for professionals, administrators and public policy makers.

The procedure established in radiology does not consist of changing the connector and syringe of the injector for each patient but exchanging only the connector with NRVs^12^. This reflects benefits for the radiodiagnosis sector, such as optimizing the time of the professionals involved and minimizing the cost of materials used during CT and MRI. However, there are no reports regarding the biosafety of this procedure and if it creates a risk for microorganism contamination in the infusion system. In this way, further studies on the safety of connectors with NRVs are required.

The objective of this study was to investigate the operation of connectors with NRVs in extreme situations to contribute to the biosafety and provide contamination and infection risk controls for infusion systems in radiological examinations. In addition, the safety of NRVs was clinically tested by evaluating the bacteriological contamination of the infusion system attached to the contrast injector. The null hypothesis is that the use of connectors with NRVs does not influence the contamination of the radiological contrast infusion system.

## Materials and Methods

**Figure 1:**
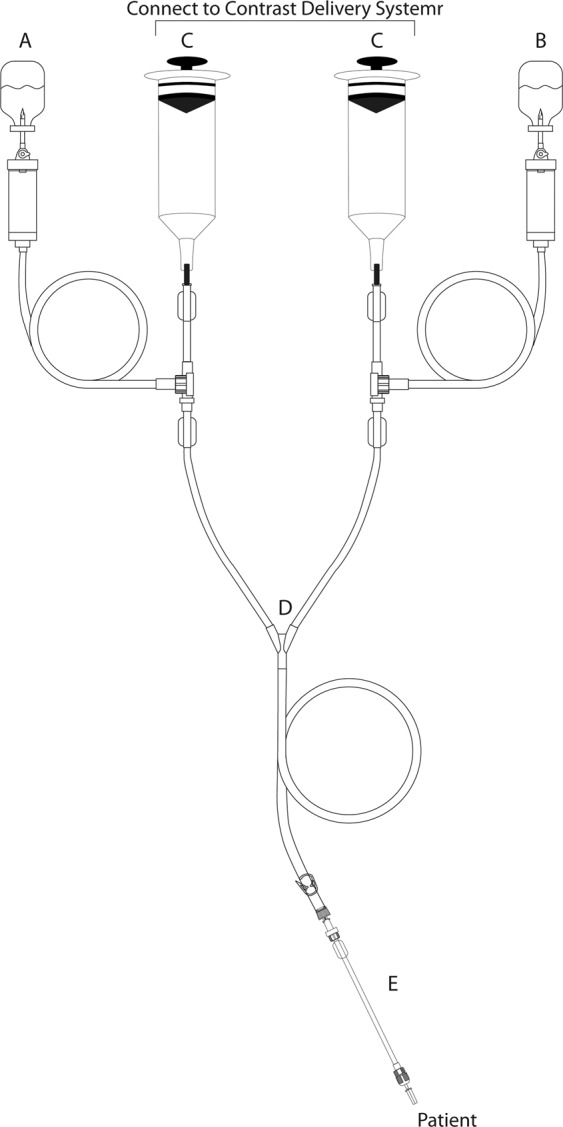
Representative scheme of the infusion system coupled to the contrast injector. (**A**) contrast container; (**B**) physiological solution container; (**C**) syringes; (**D**) contrast and physiological solution containers connectors; (**E**) connectors with NRVs.

Two hundred fifty-six connector samples (20 cm) with non-return valves (NRVs) (*Patient-set*, Alko do Brasil Indústria e Comércio Ltda., Rio de Janeiro, RJ, Brazil – batch: P07230215) were used in the study. Figure 1 shows the infusion system coupled to the contrast injector.

### Disruption evaluation of the flexible diaphragms

One hundred samples were connected to a Hydraulic Burst-Leak Tester (HB-LT – Crescent Design, San Diego, CA, USA) device during backflow to fill the extender with water. The fill rate was 4.00 cm^3^/s until the maximum pressure (1200 psi) was reached or until disruption of the flexible diaphragms (FDs).

### Functionality evaluation of the connectors with NRVs after the disruption of flexible diaphragms

Disrupted samples were divided into two experimental subgroups (A and B). Subgroup A (n = 50) was again submitted to HB-LT to evaluate the maximum pressure during backflow of disrupted FDs. In subgroup B (n = 50), the samples were connected to an air compressor (10 psi; 30 s) (Mega air – CFA-5.5/6 L, Ferrari, Cotia, SP, Brazil) to verify if there were failures in their functionality during backflow based on the release of air bubbles in a beaker with 500 mL water. Next, these samples were opened to identify, by visual inspection, the shape and integrity of the NRVs and FDs.

### Displacement analysis of the air columns in connectors with non-return valves

Fifty samples were filled with a volume of water, followed by an air column and another volume of water. The samples were connected to an air compressor (10 psi; 30 s) to inspect the movement of air columns interposed by water during backflow. The displacement (cm) and the possibility of liquid passing through the NRVs were evaluated. The procedure was repeated after the disruption of the FDs.

### Crystal violet diffusion measurements in connectors with non-return valves

Fifty multiway units (Embramed, São Paulo, SP, Brazil) were used for artificial simulations of human blood pressure. Each unit was connected to a polyurethane tube (PU – 0.8 × 50 mm) – (Autoval, Válvulas e Equipamentos Indústria Ltda., Ribeirão Preto, SP, Brazil). The units were filled with 1 mL crystal violet (1%) (Merck, St. Louis, MO, USA) and clamped. The NRV connectors were filled with 1 mL saline solution (0.85%), and one of the opposite ends of each PU tube was connected to the device. Each flow regulator (ESA 08 – Autoval) connected the PU tube to the air compressor (10 psi) in the infusion system (flow regulator, PU tube, multiway unit and connectors with NRVs). After 30 s, the flow regulator valve was closed, the compressor was disconnected, and the system was maintained at 10 psi pressure for 2 h 30 min. Then, the flow regulator was opened, and crystal violet diffusion was verified.

Each connector was cut into 10 fragments of 1 cm each from the male end of the extender with the NRV, and 30 μL aliquots of the lumen suspension were collected and transferred to a 96-well polystyrene flat-bottom plate (Thermo Fisher Scientific, Waltham, MA, USA). The readings were made in a spectrophotometer (Multiskan GO^®^, Thermo Fisher Scientific) at a wavelength of 570 nm.

### Evaluation of NRVs as a barrier to bacteriological contamination

An exponentially growing culture of methicillin-resistant *Staphylococcus aureus* (MRSA, ATCC 43300) was obtained in Mueller-Hinton Broth (BD Difco, Sparks, MD, USA) after incubation at 37 °C for 24 h. The bacterial inoculum was prepared at ~10^8^ CFU/mL according to an optical density reading in a spectrophotometer.

Fifty infusion systems (flow regulator, PU tube, multiway unit, connectors with NRVs and syringe) were used to evaluate the efficiency of the connectors with NRVs as bacteriological barriers during backflow. The multiway units were filled with 1 mL Mueller-Hinton Broth inoculated with MRSA (~10^6^ CFU/mL) to simulate bloodstream bacterial contamination above extreme cases of sepsis as reported by scientific literature from 10^3^ to 10^4^ CFU/mL^13,14^ and clamped. The NRV connectors were filled with 1 mL saline solution. Next, one end of each PU tube was connected to the device coupled to a 20 mL syringe (Becton Dickinson, São Paulo, SP, Brazil) filled with 10 mL Fluid Thioglycollate Medium (BD Difco, Sparks, MD, USA). Each flow regulator connected the PU tube to the air compressor (10 psi). After 30 s, the flow regulator valve was closed, the compressor was disconnected, and the system was maintained at 10 psi pressure for 2 h 30 min. Then, the flow regulator was opened, the syringe was decoupled, and the culture medium was incubated at 37 °C for up to 14 days and evaluated daily for turbidity. Confirmation of MRSA contamination was performed after seeding the samples on Mannitol Salt Agar (BD Difco, Sparks, MD, USA). The connectors with NRVs, damaged FDs and MRSA inoculum in the multiway unit were used as positive controls (n = 3), and those with no bacterial inoculum were used as negative controls (n = 3).

### Clinical study about the bacteriological contamination from syringes

Ninety-nine samples [saline solution (n = 49), paramagnetic contrast (n = 25) and iodine contrast (n = 25)] were collected from the syringes (Alko do Brasil Indústria e Comércio Ltda., Rio de Janeiro, RJ, Brazil – Fig. 1C) attached to connectors (*Transferfill*, Alko do Brasil Indústria e Comércio Ltda., Rio de Janeiro, RJ, Brazil – Fig. 1D) attached to the contrast (Fig. 1A) and physiological solution containers (Fig. 1B) from seven different radiological institutions. One milliliter of each solution was cultivated in 1 mL Tryptic Soy Broth (BD Difco, Sparks, MD, USA) and incubated at 37 °C for 14 days. The presence of bacterial growth was observed daily.

In the institutions, an average of 21 radiological examinations were performed with syringes (Fig. 1C) that remained attached to the connectors (Fig. 1D) to the contrast injector for up to eight hours. It is noteworthy that for each patient, only the connector with NRVs was replaced.

### Statistical analysis

Data were submitted to IBM SPSS 20.0 (IBM Corp Armonk, NY, USA) software and analyzed by descriptive statistics. Displacement of the air column before and after disruption was compared by paired t-test (significance level α = 5%). Based on the standard deviation of the difference in the response of matched pairs (0.4244) and a difference in population means (0.304), a statistical power test was applied to ensure that the sample size was sufficient to detect true differences through a statistical test. The statistical power test indicated a probability of 0.998. By convention, 0.8 is often considered an acceptable threshold.

## Results

Table 1 shows the results obtained in the applied physical tests.

**Table 1:** Maximum pressure (psi) and air bubble release (cm) of the intact and disrupted flexible diaphragms (FDs).

	**Mean** ± **SD**	**Minimum**	**Maximum**
**FD maximum pressure (psi)**			
Intact	595.44 ± 39.38	461	710
Disrupted	90.22 ± 31.26	0	135
**Air bubble release (cm)**			
Intact	0.87 ± 0.27	0.40	1.90
Disrupted	0.78 ± 0.34	0.10	1.50

SD: standard deviation.

### Disruption evaluation of the flexible diaphragms

The flexible diaphragm (FD) disruption pressures of the non-return valves (NRVs) were, on average, 148 times higher than the maximum blood pressure of 4.25 psi^15^.

### Functionality evaluation of NRVs after the disruption of flexible diaphragms

In subgroup A, among the 50 samples re-evaluated regarding the maximum backflow pressure, there was an 85% reduction in the maximum pressures supported by FDs after disruption. Two samples showed no resistance to water passage (0 psi).

**Figure 2:**
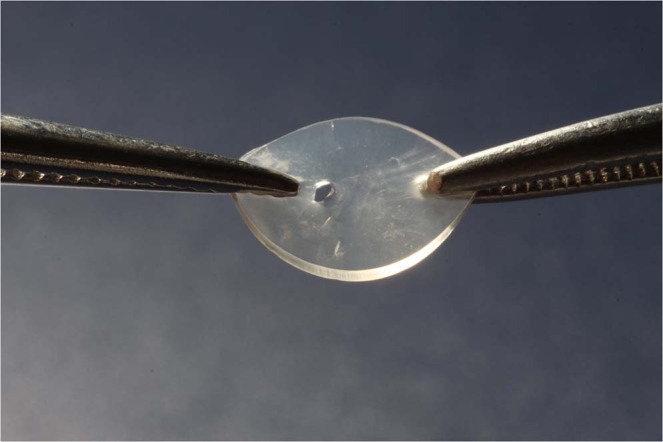
Disrupted flexible diaphragm.

In subgroup B, none of the 50 samples from disrupted FDs showed failures in NRV functionality based on the lack of air bubbles released in a beaker with water. After valve opening, lateralized FD disruption was observed, with no other deformations in the system (Fig. 2).

### Displacement analysis of the air columns in connectors with non-return valves

There were no differences among the distances traveled by air columns in NRVs with intact and disrupted FDs (p = 0.159) submitted to 10 psi per 30 s pressure.

### Crystal violet diffusion measurements in connectors with non-return valves

**Figure 3:**
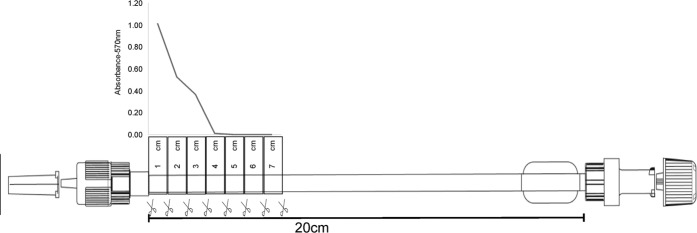
Absorbance values and diffusion distance (cm) of crystal violet measured by a spectrophotometer tended to be zero starting at 4 cm and zero at 7 cm, for a total of 20 cm.

Among the 50 analyzed samples, in 25 (50%), there was no crystal violet diffusion. The longest diffusion distance of crystal violet was 6.0 cm (1 sample, 2%). In total, 8 (16%), 8 (16%), 7 (14%) and 1 (2%) samples presented the following diffusion distances: 1.0 cm, 2.0 cm, 3.0 cm and 5.0 cm, respectively. Figure 3 illustrates the absorbance values and diffusion distances of crystal violet.

### Evaluation of NRVs as a barrier to bacteriological contamination

None of the 50 samples from the infusion system or the negative controls presented bacterial growth. There was isolation of MRSA only in the positive controls.

### Clinical study about the bacteriological contamination from syringes

Bacterial growth was not observed in any of the evaluated samples from seven different radiological institutions that employed NRVs for biosafety in their clinical practice and as contamination and infection risk controls in infusion systems in radiology.

## Discussion

The use of connectors with non-return valves (NRVs) aims to reduce the risk of contamination during imaging tests and provide more safety in radiology. Nevertheless, the scientific literature shows few studies^7,12,16–22^ on connectors with NRVs employed in health.

In this study, we sought, in extreme situations, to explore the concerns regarding the operation of connectors with NRVs. The null hypothesis was denied since it was observed that the use of connectors with NRVs is essential to preventing contamination of the radiological contrast infusion system.

According to Whelton *et al*.^15^, human blood pressure is classified as normal when the systole value is less than 120 mmHg (2.32 psi) and the diastole value is less than 80 mmHg (1.54 psi), and it is classified as extreme when the systole value reaches 220 mmHg (4.25 psi). Thus, even in extreme cases, the maximum blood pressure (4.25 psi) would remain far below that required to break the flexible diaphragms (FDs) of connectors with NRVs (mean and standard deviation: 595.44 ± 39.38 psi) or widen the cracks of disrupted FDs from NRVs (mean and deviation standard: 90.22 ± 31.26 psi). The air compressor used to conduct these tests provided a safety factor that exceeds human blood pressure.

The evaluation of the structure and integrity of disrupted FDs from NRV connectors identified the presence of cracks in the FDs; however, the other NRV structures remained intact. Therefore, it was inferred that NRV failures could occur due to inadequate FD positioning in the bulkheads; nevertheless, backflow (10 psi; 30 s) was not able to cause failures in NRVs, even with disrupted FDs.

Air columns can travel through NRV connectors with intact (up to 1.90 cm) and disrupted (up to 1.50 cm) FDs. In this way, professionals must completely fill the connectors with NRVs with physiological solution to prevent air bubbles and/or air columns from migrating through the connectors. Bloodstream infection prevention is especially important in this regard, since the displacement of such columns could disseminate microorganisms present in the bloodstream through the connectors with NRVs.

In this study, crystal violet was chosen because of the ease of detecting it by spectrophotometry, while Vermeulen *et al*.^12^ used Patent Blue V^®^, and Nandy *et al*.^19^ used bromophenol blue and food colorings as well as radiolabels. Regarding the dye diffusion experiment through connectors with NRVs, the pressure employed was also 10 psi, but for 2 h 30 min. This period was determined by doubling the value of the maximum time spent (~1 h 15 min) to perform all preparations needed for a radiological clinical procedure (~50 min and wait time of ~25 min). This time is different from that in previously published works, which varied from 15 s to 72 h^7,12,16–19^. However, it is necessary to define a longer time based on clinical practice to optimize the safety factor.

The effectiveness of NRVs during backflow as a barrier to bacterial contamination was evidenced by the absence of MRSA growth. Other studies investigated the risks for cross-contamination *in vitro* and *in vivo* of different NRVs used in contrast injection devices in radiology. Such a result shows that connectors with NRVs reliably prevented fluid backflow and work as a barrier to microorganisms. Similar to the results found in this study, other authors^7,12,18,20–22^ have not found evidence of the passage of microorganisms through connectors with NRVs. On the other hand, other authors^16,17,19^ have demonstrated contamination of the system. The discrepancy between these results can be explained by the use of different materials, types and models of NRVs (springless: Merit Medical System^®^ and Namic^16^; made of latex: Braun Melsungen^®^, Braun Spezial^®^, Infudrop^®^, Becton-Dickinson^®^ and Smith-Medical^®^^17^; the valve design varies slightly between the five models and may be designed with a free-floating disk guided by the valve housing – valve C, or a diaphragm attached in some way to either the inflow side – valve E or outflow side of the valve – valves A, B, and D^19^). Furthermore, different methodologies (60 psi^16^ and 2 mL/h^17^ during backflow with syringe pump and 4.64 psi during backflow with arthroscopy injector^19^) and diffusion times (up to 1 h^16^, 24 h^19^ and 72 h^17^) were reported.

In this study, no functionality failures were observed in connectors with NRV (Alko do Brasil Indústria e Comércio Ltda., Rio de Janeiro, RJ, Brazil) through the applied tests (at 10 psi pressure for 2 h 30 min). Therefore, it was believed that safety in device usage also depends on the technique and expertise of health/nursing professionals.

Both within the community and health institutions, the hands represent the main vehicle for microbial dissemination. Thus, hand hygiene (HH) is the simplest and most effective way to prevent healthcare-associated infections (HAIs)^23–26^. The increase in the number of hospitalization days for patients due to HAIs can be controlled and/or reduced by implementing adequate programs promoting hand hygiene for health professionals, which leads to a great reduction in financial costs^25^.

The safety of NRVs in radiology infusion systems is not yet a consensus and depends on various physical, chemical, microbiological factors and the proper handling of connectors and syringes by health professionals.

In addition, it is essential to highlight that syringes usually remain attached to the system for eight hours, and well-designed and technically rigorous clinical procedures contribute to the safety of NRVs in clinical practice.

The bacterial contamination in contrast syringes with connectors without NRVs has already been reported in a study^20^. Nevertheless, our clinical study did not showed bacteriological contamination from ninety-nine syringes [saline solution (n = 49), paramagnetic contrast (n = 25) and iodine contrast (n = 25)] (Alko do Brasil Indústria e Comércio Ltda., Rio de Janeiro, RJ, Brazil – Fig. 1C) attached to connectors (*Transferfill*, Alko do Brasil Indústria e Comércio Ltda., Rio de Janeiro, RJ, Brazil – Fig. 1D) attached to the contrast (Fig. 1A) and physiological solution containers (Fig. 1B) from seven different radiological institutions, corroborating the studies that also used connectors with NRVs and did not show bacterial contamination in contrast syringes^21,22^.

In the institutions of our study, an average of 21 radiological examinations were performed with syringes (Fig. 1C) that remained attached to the connectors (Fig. 1D) attached to the contrast injector for up to eight hours. It is noteworthy that for each patient, only the connector with NRVs was replaced.

Bacterial contamination and biofilm formation are remarkable issues in HAIs. Consistent with the results observed *in vitro*, in the clinical study, bacterial contamination was not observed in the syringes coupled to the contrast and physiological solution containers. This means that the connectors with NRVs are safe as bacteriological barriers to backflow, even though the syringes remained connected to the system for up to eight hours.

The limitations of this study are those inherent to *in vitro* studies. However, the extrapolation of these results to clinical situations depends on different situations related to the behaviors of professionals and patients, following the recommendations of the standard operating procedure for the use and handling of connectors with NRVs employed in this study.

In summary, testing the functionality and safety of NRVs in terms of contamination and infection risks in infusion systems in radiology should be part of the evaluation of this medical device liberation that is not a regulatory requirement nowadays. Moreover, we emphasize the importance of developing a study of technical and economic viability that demonstrates the contribution of the use of these medical devices to infection control and, consequently, reduction of costs related to the management of HAIs.
